# Mechanistic insights into poly(C)-binding protein hnRNP K resolving i-motif DNA secondary structures

**DOI:** 10.1016/j.jbc.2022.102670

**Published:** 2022-11-02

**Authors:** Wen-Qiang Wu, Xin Zhang, Di Bai, Song-Wang Shan, Li-Jun Guo

**Affiliations:** School of Life Sciences, Academy for Advanced Interdisciplinary Studies, State Key Laboratory of Crop Stress Adaptation and Improvement, School of Physics and Electronics, Henan University, Kaifeng, China

**Keywords:** hnRNP K, i-motif, smFRET, *c-MYC*, oncogene, cancer, hnRNP K, heterogeneous nuclear ribonucleoprotein K, PCBP, poly(C)-binding protein, smFRET, single-molecule FRET, TDP, transition density plot

## Abstract

I-motifs are four-strand noncanonical secondary structures formed by cytosine (C)-rich sequences in living cells. The structural dynamics of i-motifs play essential roles in many cellular processes, such as telomerase inhibition, DNA replication, and transcriptional regulation. In cells, the structural dynamics of the i-motif can be modulated by the interaction of poly(C)-binding proteins (PCBPs), and the interaction is closely related to human health, through modulating the transcription of oncogenes and telomere stability. Therefore, the mechanisms of how PCBPs interact with i-motif structures are fundamentally important. However, the underlying mechanisms remain elusive. I-motif structures in the promoter of the *c-MYC* oncogene can be unfolded by heterogeneous nuclear ribonucleoprotein K (hnRNP K), a PCBP, to activate its transcription. Here, we selected this system as an example to comprehensively study the unfolding mechanisms. We found that the promoter sequence containing 5 C-runs preferred folding into type-1245 to type-1234 i-motif structures based on their folding stability, which was further confirmed by single-molecule FRET. In addition, we first revealed that the *c-MYC* i-motif structure was discretely resolved by hnRNP K through two intermediate states, which were assigned to the opposite hairpin and neighboring hairpin, as further confirmed by site mutations. Furthermore, we found all three KH (hnRNP K homology) domains of hnRNP K could unfold the *c-MYC* i-motif structure, and KH2 and KH3 were more active than KH1. In conclusion, this study may deepen our understanding of the interactions between i-motifs and PCBPs and may be helpful for drug development.

In addition to duplex type, DNA can adopt some noncanonical structures, such as four-stand structure G-quadruplexes (1) and i-motifs (2). Among these two structures, G-quadruplexes that form within guanine (G)-rich sequences have been well studied from many different perspectives (1, 3, 4). However, their complementary i-motif structures are comparatively less well studied. I-motif structures are formed from cytosine (C)-rich DNA sequences at acidic pH, held together by hemi-protonated C and neutral C base pairs (C:C^+^) (2). Moreover, at neutral pH, molecular crowding can facilitate the formation of i-motif structures, which is in keeping with the intracellular environment (5). In addition, the existence of i-motif DNA structures was revealed using in-cell NMR spectroscopy (6) and confirmed by an antibody fragment (7) in cells. The structural dynamics of these motifs play key roles in many essential processes, such as telomerase inhibition, DNA replication, and transcriptional regulation (2).

The nuclease hypersensitive element III_1_ is located in the upstream of the oncogene *c-MYC* P1 promoter and modulates most *c-MYC* transcription. It contains 27-bp G/C-rich DNA sequences and can form G-quadruplexes and i-motifs *in vivo* (8, 9). In recent years, G-rich strands that form G-quadruplexes (known as Pu27) have been well studied (9). Although the complementary C-rich strands (referred to as Py27) that form i-motifs can also modulate *c-MYC* expression (8), they are comparatively less well studied. The core of Py27 (termed Py25 in this study) contains 5 runs of cytosines: C-tracts 1, 2, 3, 4, and 5 (Fig. 1*A*). Whether type-1245 (10) or type-1234 (11) is the major structure is the subject of controversy (Fig. 1*B*).

**Figure 1:**
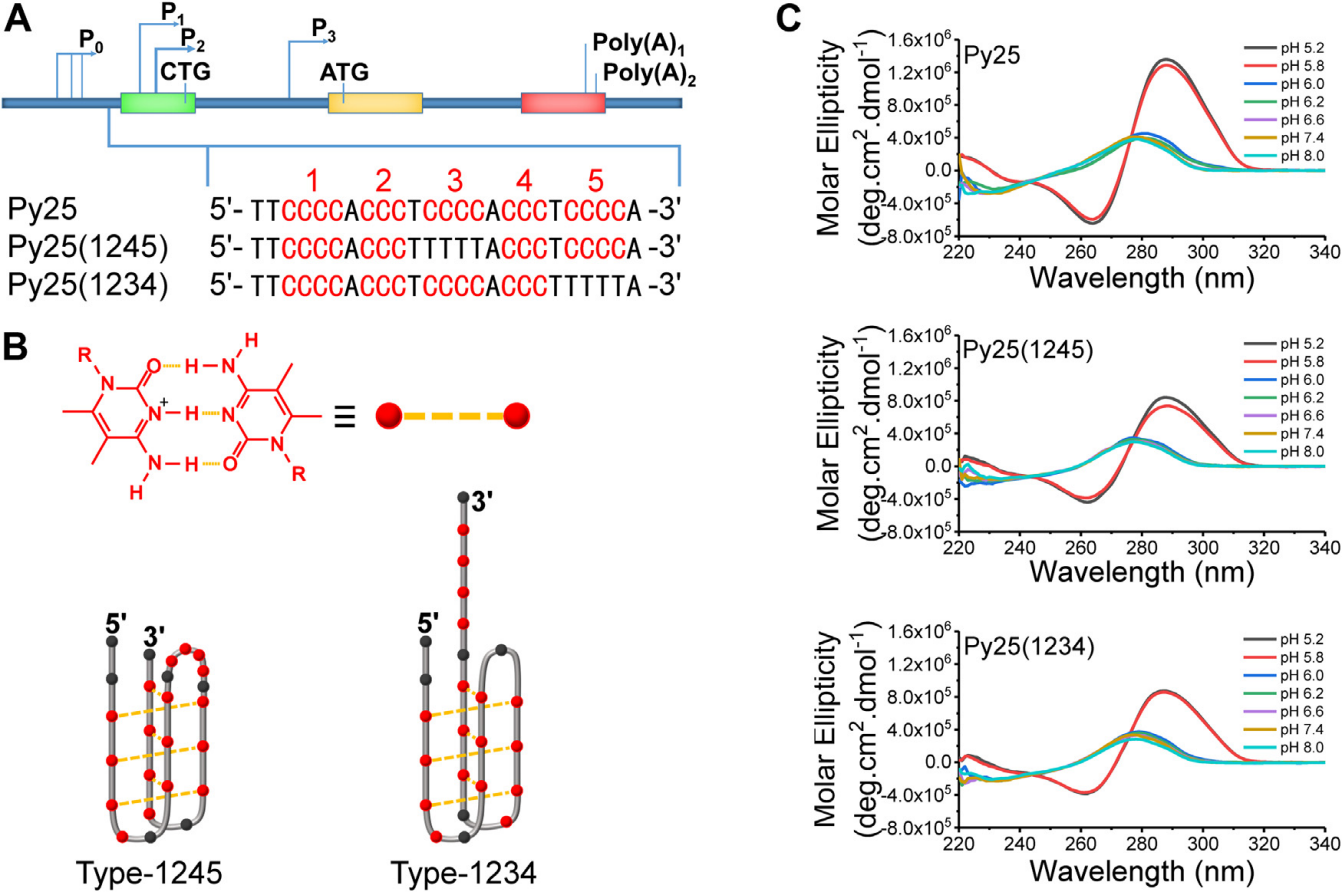
**Py25 and its modifications can fold into i-motif structures.***A*, the promoter sequences of NHE III_1_ and its modifications. Py25 indicates the 25-nt core of the C-rich NHE III_1_ promoter containing 5 runs of cytosines, as marked in *red*. Py25(1245) and Py25(1234) contain four runs of cytosines, which are indicated in the parentheses. *B*, a schematic representation of the C:C^+^ base pair (*top*) and the topological structures of type-1245 and type-1234 (*bottom*). *C*, CD spectra of Py25, Py25(1245), and Py25(1234) at different pH levels from 5.2 to 8.0, including 200 mM K^+^, 40% PEG200 (w/v), and 4 mM Trolox. Under pH 5.8, all three substrates could fold into i-motif structures, showing a positive peak at ∼287 nm and a negative peak at ∼262 nm. NHE, nuclear hypertensive element.

The structures of i-motifs are dynamic in living cells (7), and their structural dynamics regulate biological processes (2). Although the structural dynamics of i-motifs induced by environmental conditions such as pH and PEG have been studied using both bulk and single-molecule methods (12, 13, 14, 15), their dynamics in cells are mainly modulated by i-motif specialized proteins (2). Considering that i-motifs can exist stably in cells (6, 7), the unwinding of these structures is particularly critical. However, at this stage, little is known about their unfolding pathways facilitated by biologically specialized binding proteins, such as hnRNP LL (16), hnRNP A1 (17), BmILF (18), and poly(C) binding proteins (PCBPs) (19). Among the various proteins, heterogeneous nuclear ribonucleoprotein K (hnRNP K), a PCBP, stands out for its extensive cellular activity. This protein can activate the transcription of oncogenes though unfolding their promoter i-motifs, such as *KRAS* (20) and *c-MYC* (21, 22), and may modulate telomere stability by binding telomeric i-motifs (23, 24). However, the molecular mechanism of how hnRNP K unfolds i-motif structures remains elusive due to the limitations of research methods.

hnRNP K contains three KH (hnRNP K homology) domains (Fig. S1*A*) that mediate the resolution of i-motif structures (25, 26, 27). Previous structural data have shown that KH3 binds specifically to TCCC or CCCC ssDNA (25, 26). Biochemical assays have indicated that the KH1–KH2 domain can also bind poly(C) sequences (27). However, it remains unclear whether all three of these motifs can disrupt i-motif structures and which motif exhibits the most activity. Studying the interaction of hnRNP K with i-motifs will not only provide helpful data for understanding this biological process and designing drugs but also be useful for predicting the molecular mechanisms of KH domain containing PCBPs (19, 28).

In this study, hnRNP K was selected as an example to dissect the molecular mechanism of PCBP-unfolding i-motif structures. First, CD spectra were used to detect the folding of Py25, Py25(1245), and Py25(1234) under different pH conditions. Melting temperature comparison suggested that the type-1245 i-motif should be the major component of Py25, which was further confirmed by single-molecule FRET (smFRET). Then, it was confirmed that hnRNP K could resolve the Py25 i-motif using an ensemble FRET method, and the folding intermediates and pathways were determined using smFRET. Finally, it was revealed that all three of the KH domains were able to unfold Py25 i-motif structure, and KH2 was the most active. This study sheds light on the interactions between PCBPs and i-motif structures.

## Results

### Type-1245 is the main i-motif structure of Py25

Structure determines function, so it is fundamentally important to determine the folding structure of Py25 to reveal its function. The G-rich strand of the *c-MYC* promoter that forms G-quadruplexes has been well studied, and type-2345 and type-1245 *c-MYC* G-quadruplexes are two major structures (29, 30). Therefore, complementary type-1234 and type-1245 i-motifs were proposed to be the major folding structures; however, whether type-1245 or type-1234 is the main structure remains controversial (10, 11). To determine whether type-1245 or type-1234 was the major folding structure (Fig. 1*B*), the unrelated C in Py25 was mutated to T to rule out distractions, and they were referred to as Py25(1245) and Py25(1234), respectively (Fig. 1*A*). Their folding was determined at different pH levels (Fig. 1*C*), as pH was the main factor affecting the stability of i-motifs (15). The CD spectra of Py25, Py25(1245), and Py25(1234) showed that at pH ≥ 6.0, there were no obvious i-motif structures, while when at pH ≤ 5.8, a significant i-motif signal was detected, showing a positive peak at ∼287 nm and a negative peak at ∼262 nm (5). These results suggested that both type-1245 and type-1234 might form i-motif structures at pH values below 5.8.

**Figure 2:**
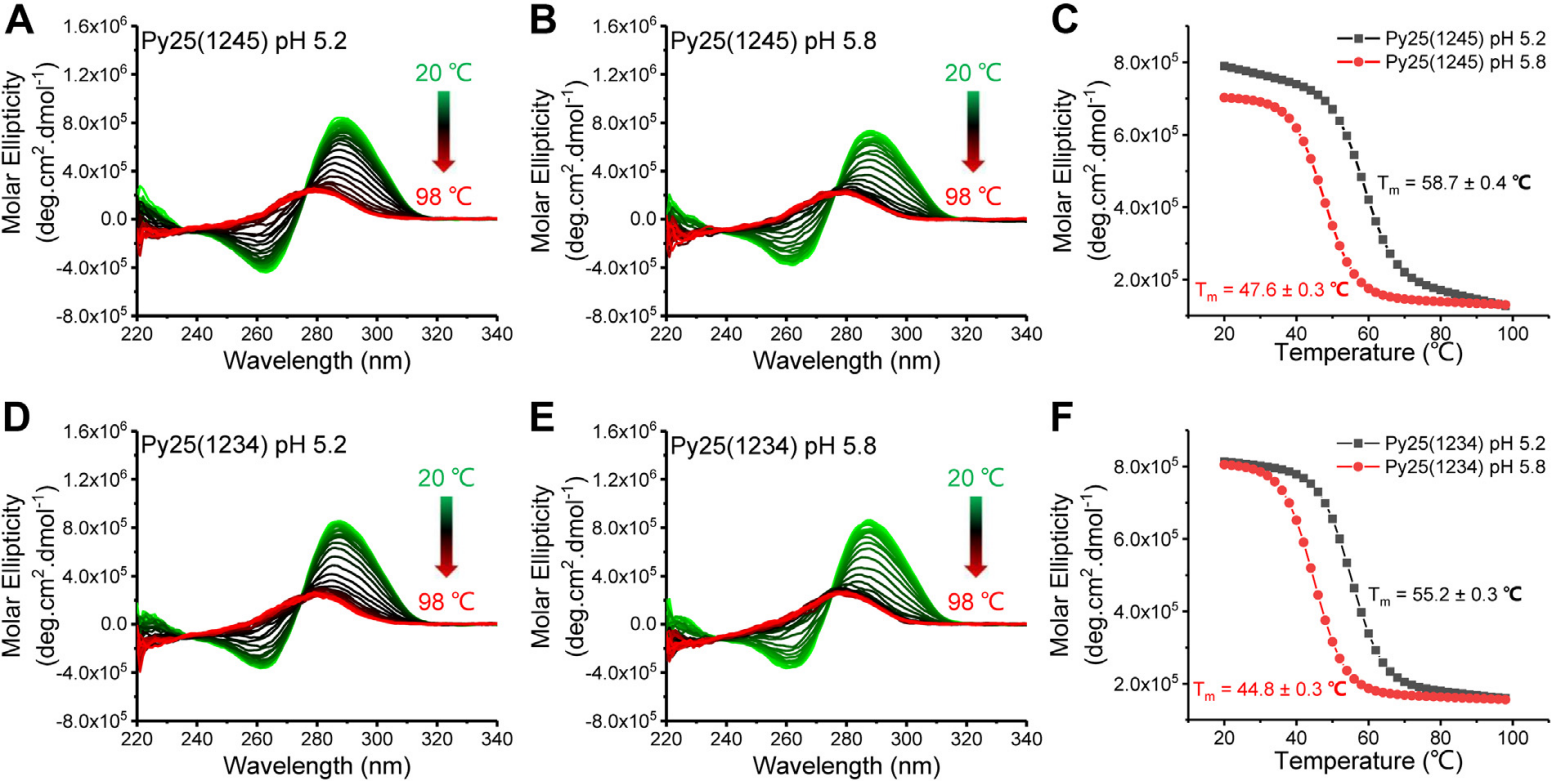
**CD melting of Py25(1245) and Py25(1234) at pH 5.2 and pH 5.8.***A*–*C*, the CD spectra of Py25(1245) at 20 to 98 °C at pH 5.2 (*A*) and pH 5.8 (*B*). Melting temperature (*T*_m_) values are calculated from the signal at 288 nm, resulting in the *T*_m_ values of 58.7 ± 0.4 °C for pH 5.2 and 47.6 ± 0.3 °C for pH 5.8. *D*–*F*, the CD spectra of Py25(1234) at 20 to 98 °C at pH 5.2 (*D*) and pH 5.8 (*E*). *T*_m_ values were calculated from the signal at 288 nm, resulting in the *T*_m_ values of 55.2 ± 0.3 °C for pH 5.2 and 44.8 ± 0.3 °C for pH 5.8.

To determine whether type-1245 or type-1234 was the main structure, this study first measured their melting temperature (*T*_m_) values using the CD spectra at pH 5.2 and pH 5.8 (Fig. 2). The comparison of *T*_m_ values demonstrated that type-1245 was more thermally stable than type-1234, because smaller *T*_m_ values reflected more unstable structures. Based on these results, the type-1245 i-motif should be the major component of Py25. This result was inconsistent with a previous report, in which the *T*_m_ value of type-1234 was greater than that of type-1245 (11). The difference between these two studies was that the present study replaced the fifth C-tract of Py25(1234) with T, while the previous study removed the fifth C-tract entirely. The proximal ssDNA of the Py25(1234) used in the present study may have reduced its stability, as in the case of G-quadruplexes (31, 32). The substrate Py25(1234) used in the present study did not change the overall length of Py25; thus, our findings are likely more biologically relevant.

**Figure 3:**
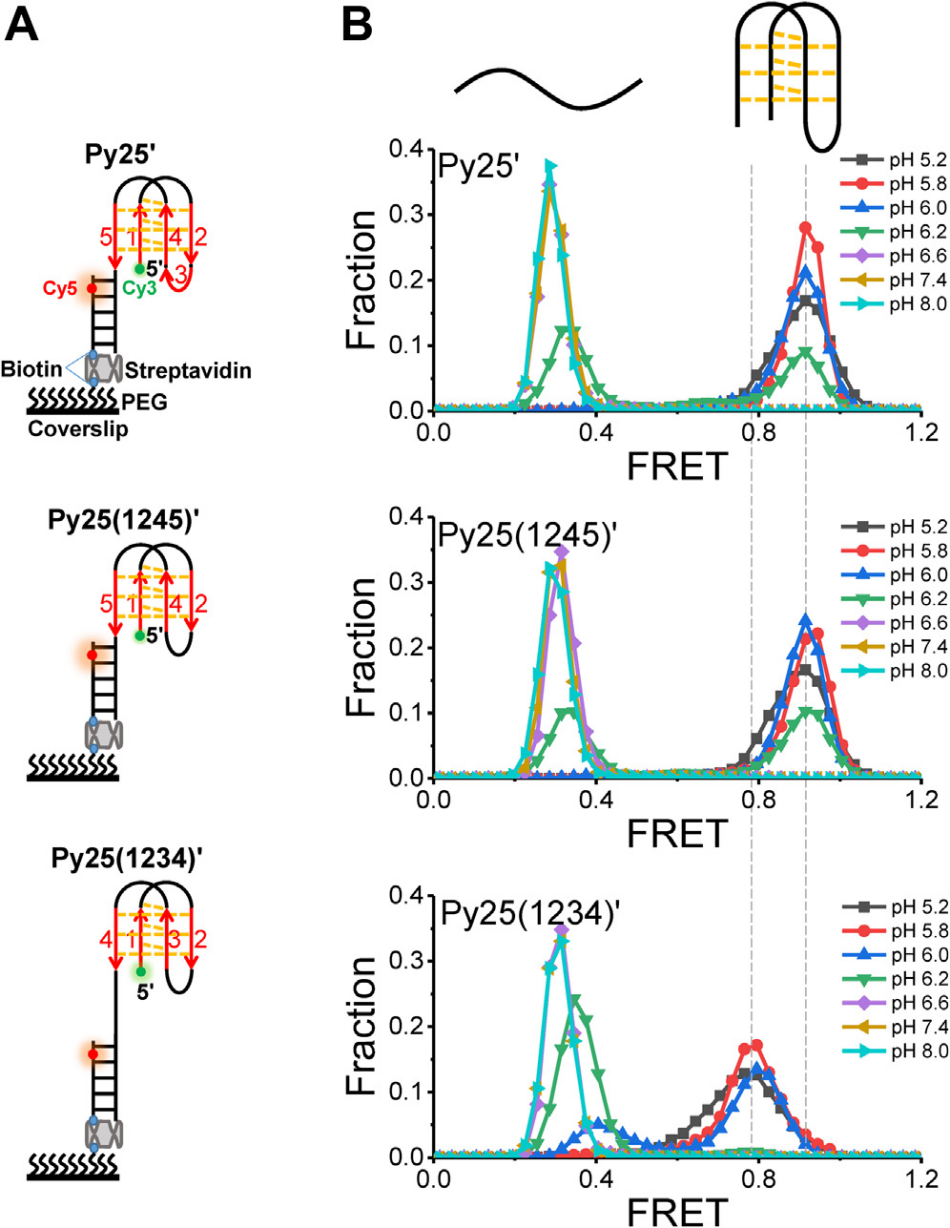
**The folding of Py25′, Py25(1245)**′**, and Py25(1234)**′ **at different pH levels from 5.2 to 8.0 using single-molecule fluorescence resonance energy transfer.***A*, schematic diagram of Py25′, Py25(1245)′, and Py25(1234)′. *B*, FRET distributions of Py25′, Py25(1245)′, and Py25(1234)′ at different pH levels. The FRET values less than 0.4 should be ssDNA, and the FRET values more than 0.8 should be unfolded i-motif DNA, according to the CD spectra in Fig. 1*C*.

To further confirm the above results, smFRET, which can be used to directly determine the folding states and intermediates of DNA secondary structures through FRET efficiency (14, 32, 33, 34), was introduced. An i-motif–containing ssDNA was modified by a Cy3 at the 5′ end, and its 3′ end was hybridized with a complementary stem strand labeled by biotin at the 5′ end and a Cy5 at the fifth nucleotide from the 3′ end. This donor (Cy3) and acceptor (Cy5) spacing can sensitively reveal the conformational change in i-motif structures *via* the FRET signal (14, 32, 33, 34). Py25, Py25(1245), and Py25(1234) containing smFRET substrates were referred to as Py25′, Py25(1245)′, and Py25(1234)′, respectively (Fig. 3*A*). The biotin was used to immobilize the substrates on PEG-modified surface *via* streptavidin. With the decrease in pH, i-motif structures gradually folded (Fig. 1*C*), leading to the incremental increase of FRET efficiency (Fig. 3*B*) (13). Therefore, a FRET value of about 0.3 to 0.4 should correspond to the ssDNA state, and values of ∼0.8 and ∼0.9 should correspond to well-folded i-motif structures, consistent with a recent report (13). Based on the principle of FRET, the further the distance is, the lower the FRET value will be. Comparing the structures of Py25(1234)′ with that of Py25(1245)′ (Fig. 3*A*), the i-motif structure in Py25(1234)′ was separated from dsDNA with ssDNA; thus, the distance between Cy3 and Cy5 in Py25(1234)′ is greater than that in Py25(1245)′, leading to a lower FRET value. Therefore, it was easy to conclude that Py25 was folded into the structure of type-1245 instead of type-1234 based on the FRET values of the folded states. In addition, the corresponding smFRET substrates Py25(1235)′, Py25(1345)′, and Py25(2345)′ were designed (Fig. S2*B*) to check the potential for type-1235, type-1345, and type-2345 folding (Fig. S2*A*), and their FRET distributions under different pH from 5.2 to 8.0 are shown in Fig. S2*C*. Based on the principle of FRET, it is expected that the FRET values of folded Py25(1235)′ and Py25(1345)′ will be similar to that of Py25′, and the FRET values of folded Py25(2345)′ will be lower than that of Py25′ because of the extra ssDNA tail. The experimental FRET values were in agreement with this expectation. From the folding fractions, it was clear that Py25(1245)′ folding was significantly better than that of other mutants (Fig. S2*D*). In addition, like Py25(1234)′, Py25(2345)′ was ruled out again based on its lower FRET values than that of Py25′ (Figs. 3*B* and S2*C*). Therefore, we concluded that type-1245 is the most favored structure.

### Dynamics of hnPNP K unfolding Py25

**Figure 4:**
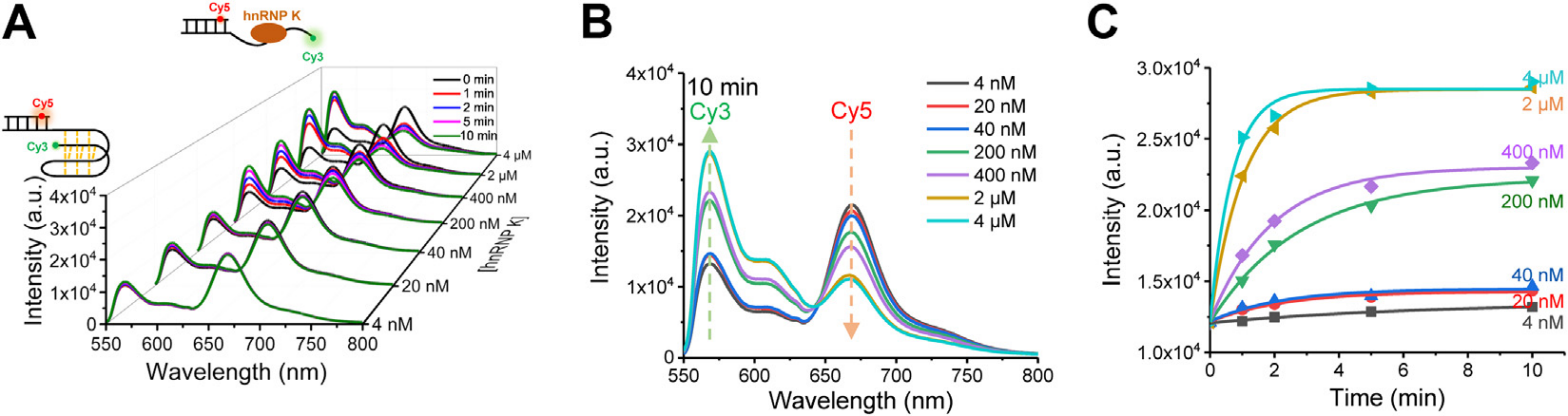
**Dynamics of hnPNP K unfolding Py25′ i-motif DNA using the bulk fluorescence resonance energy transfer method at pH 5.8.***A*, serial fluorescence emission spectra of Py25′ with increasing hnRNP K concentrations from 4 nM to 4 μM at different times from 0 to 10 min. A laser at 532 nm was used to excite Cy3. *B*, fluorescence emission spectra of Py25′ with increasing hnRNP K concentrations from 4 nM to 4 μM at 10 min. The *arrows* at 570 nm and 670 nm represent the evolution of Cy3 and Cy5, respectively. *C*, single-exponential decays were used to fit the emission of Cy3 in (*B*), resulting in the unwinding parameters shown in Table S2. hnRNP K, heterogeneous nuclear ribonucleoprotein K.

After determining the major structure of Py25, this study sought to determine how proteins in cells unfolded this structure. PCBPs have been shown to play essential roles in i-motif dynamics. Among them, hnRNP K is involved in *c-MYC* i-motif unfolding through binding to the C-rich strand (21). However, the unfolding dynamics have remained elusive. This study first expressed and purified hnRNP K to homogeneity (Fig. S1*B*). Then, the binding of hnRNP K to Py25′ was assessed using PAGE bandshift assays (Fig. S3). The apparent DNA-binding affinity value was about 220 nM. At 4 μM, almost all Py25′ was bound by hnRNP K. Therefore, concentrations below 4 μM were selected for subsequent analyses. To determine whether hnRNP K could unfold the *c-MYC* i-motif in this system at pH 5.8, Cy3 dyes were excited using a laser at 532 nm, and the bulk fluorescence spectra of Py25′ were monitored after adding different concentrations of hnRNP K from 4 nM to 4 μM (Fig. 4*A*). The emission wavelengths of ∼670 nm and ∼570 nm represented Cy5 and Cy3, respectively. It was clear that at each concentration of hnRNP K, the fluorescence spectra shifted gradually over time from ∼670 nm to ∼570 nm, resulting in the decrease of the FRET efficiency and indicating that the Py25 i-motif structure was unfolded by hnRNP K. The increase in Cy3 intensity and decrease in Cy5 intensity confirmed that the FRET efficiency decrease resulted from i-motif structure unfolding and ruled out quenching or some other mechanisms. At a certain time, such as 10 min (Fig. 4*B*), the unwinding ratio gradually increased as the hnRNP K concentration increased. Furthermore, the emissions of Cy3 at different concentrations of hnRNP K were fitted using single-exponential decays to quantify the unwinding parameters (Fig. 4*C*). It was found that the higher the concentration, the faster the unwinding (Fig. 4*C* and Table S2).

### hnRNP K unfolds Py25 i-motif structure with four discrete states

**Figure 5:**
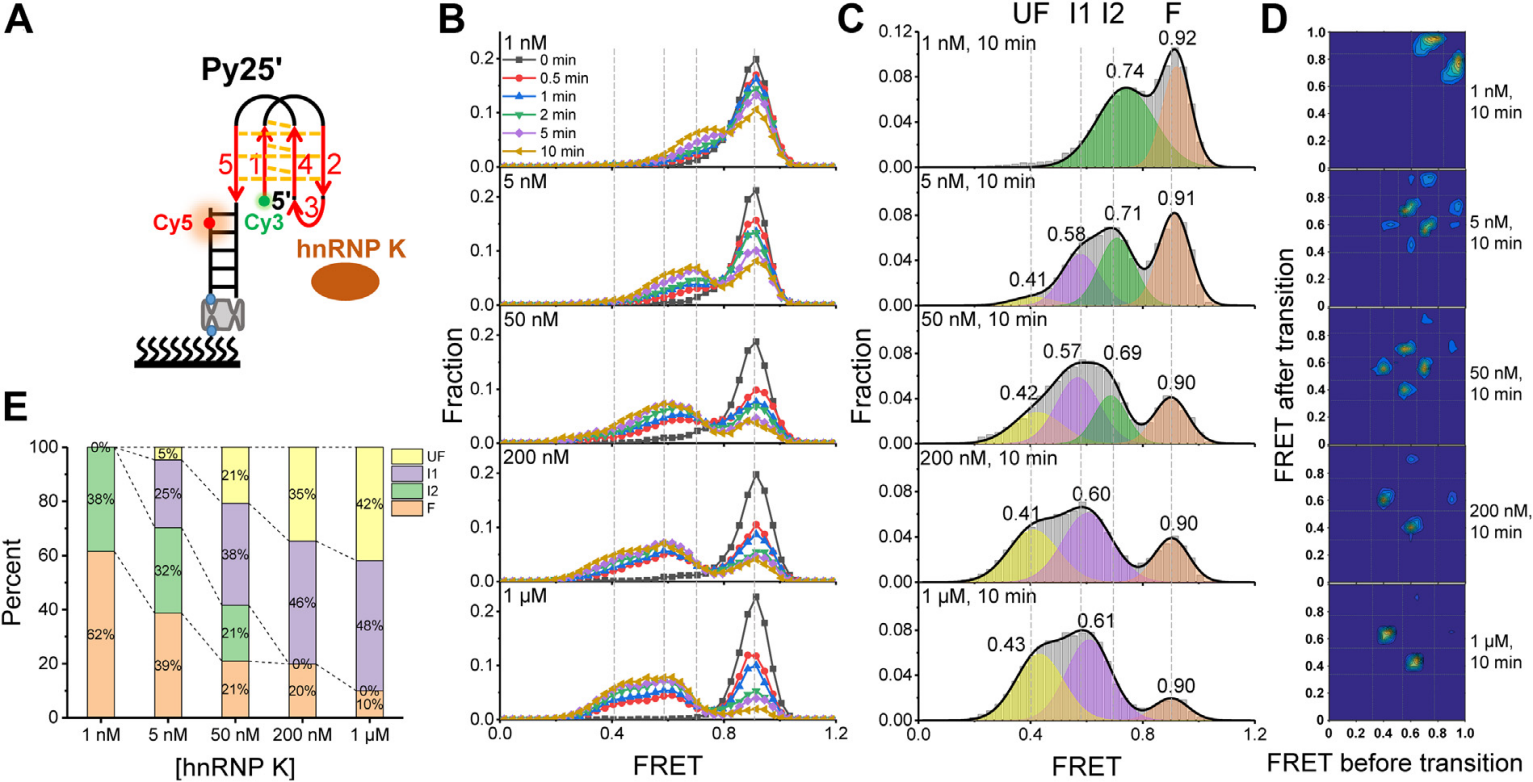
**hnRNP K unfolding Py25′ i-motif DNA discretely revealed by single-molecule fluorescence resonance energy transfer.***A*, schematic diagram of hnRNP K and Py25′ i-motif DNA. *B*, smFRET histograms obtained by adding 1 nM–1 μM hnRNP K at a series time from 0 to 10 min at pH 5.8. *C*, multipeak Gaussian distributions were used to fit the smFRET histograms at 10 min. The peak values are shown in the corresponding figures. *D*, transition density plots (TDPs) were used to show the state transitions of hnRNP K unfolding Py25′ at 10 min and certain concentrations. *E*, the fractions of the different folding structures at increasing concentrations of hnRNP K. hnRNP K, heterogeneous nuclear ribonucleoprotein K; smFRET, single-molecule fluorescence resonance energy transfer.

To further dissect the intermediates and dynamics of hnRNP K unfolding Py25 i-motif structure, the smFRET method was applied to monitor the structural changes of Py25′ in real time at varied concentrations of hnRNP K under pH 5.8 (Fig. 5*A*). With the addition of 1 nM–1 μM hnRNP K, the FRET efficiencies gradually shifted towards low FRET values over time (Fig. 5*B*), indicating that the i-motif structure was resolved. The FRET histograms at 10 min were well fitted using a multipeak Gaussian distribution (Fig. 5*C*), which is widely used to recognize dynamic states at the single molecule level (14, 32, 33, 34), resulting in 2 to 4 peaks. At 1 nM hnRNP K, two FRET value peaks of ∼0.9 and ∼0.7 were detected. At 5 to 50 nM hnRNP K, as many as four peaks of FRET values at ∼0.9, ∼0.7, ∼0.6, and ∼0.4 were captured. At 200 nM–1 μM hnRNP K, there were only three states, and the peak at ∼0.7 disappeared. The FRET peaks of ∼0.9 and ∼0.4 at pH 5.8 were assigned to complete unfolded (UF) and folded (F) states (Figs. 1*C* and 3*B*). Therefore, the two middle states should be the folding intermediates (referred to as I1 and I2 in Fig. 5*C*). Furthermore, transition density plots (TDPs) were constructed to directly show the state transitions between these states (Fig. 5*D*). Their representative traces are shown in Fig. S5*A*, and their proportions are shown in Figure 5*E*. As the protein concentration increased, the fully folded proportion gradually decreased and the unfolded proportion gradually increased.

To further confirm whether Py25′ and Py25(1245)′ folded into the same i-motif structure, this study investigated the interaction between hnRNP K and Py25(1245)′ under the same experimental conditions (Fig. S4*A*). As expected, the phenomena, states, and distributions were similar to those of hnRNP K unfolding Py25′ (Figs. S4 and S5*B*). One small difference was that hnRNP K resolved Py25′ more efficiently than it unwound Py25(1245)′ (Figs. 5*E* and S4*E*). This difference may have been caused by the third C-tract; hnRNP K may bind to this CCCC tract (25, 26) to accelerate the disruption of the i-motif structure.

### hnRNP K resolves i-motif DNA with opposite and neighboring hairpins as intermediate states

**Figure 6:**
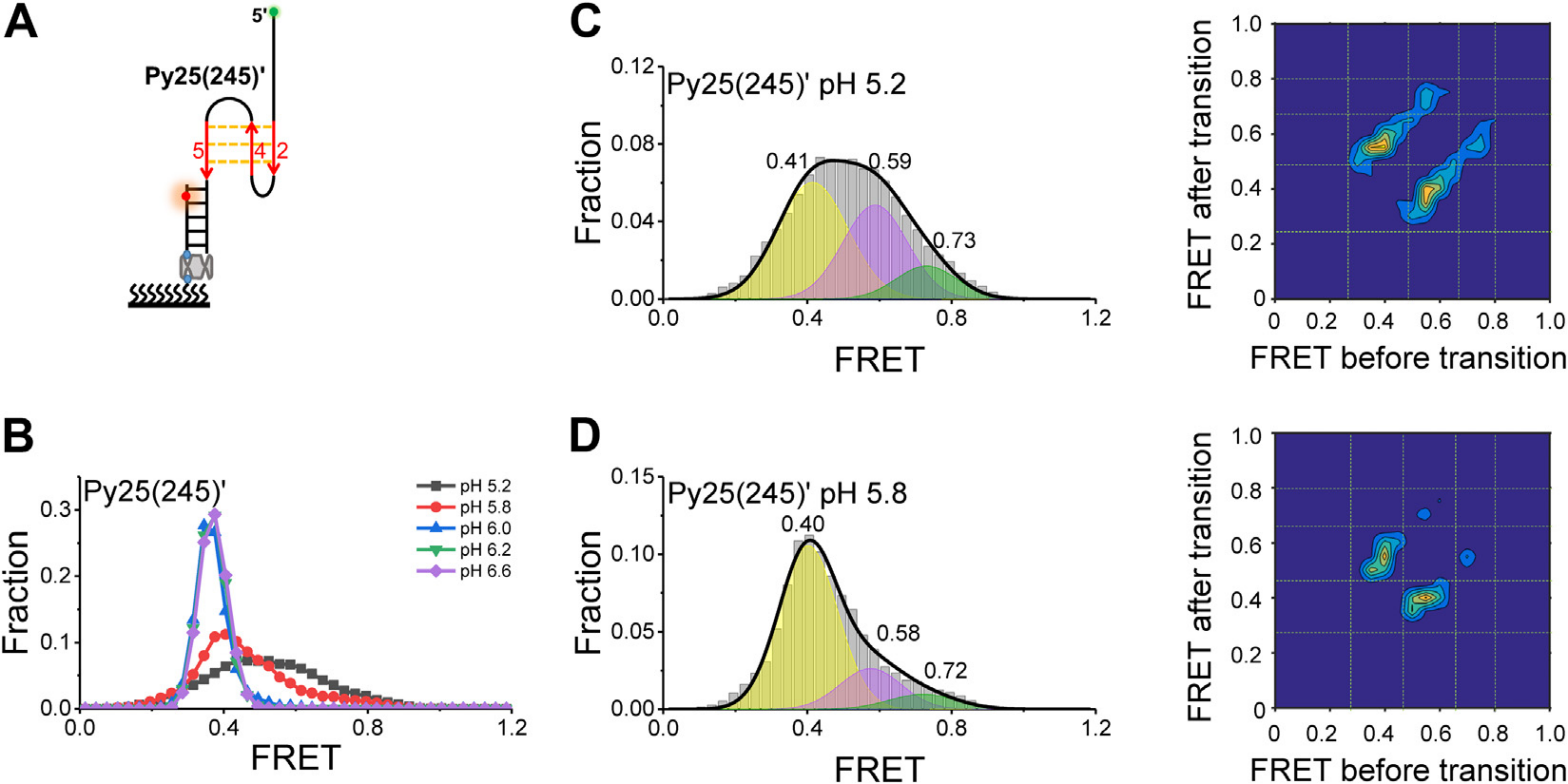
**Formation of opposite C-hairpins at pH 5.2 and pH 5.8.***A*, schematic view of Py25(245)′. *B*, FRET distributions of Py25(245)′ at different pH values. *C* and *D*, single-molecule FRET (smFRET) histograms of Py25(245)′ at pH 5.2 (*C*) and pH 5.8 (*D*) were fitted by multipeak Gaussian distributions (*right panel*), and three peaks were detected for each histogram. TDPs were used to show their state transitions (*left panel*). TDP, transition density plot.

Next, this study sought to determine which structures the intermediate states corresponded to. Based on the aggregation of i-motifs (Fig. 1*B*), it could be concluded that the intermediate states should represent the disruption of partial hydrogen bonds. The KH domain can stably bind one C-column (25, 26, 28). Therefore, it was hypothesized that the intermediates should be different kinds of C-hairpins. The i-motif structure consisted of two opposite hairpin structures (Fig. 1*B*). At a low hnRNP K concentration (1 nM), only one more state ∼0.7 appeared (Fig. 5, *B* and *C*, top panel). It was speculated that the ∼0.7 state should be an opposite hairpin, resulting from the binding of hnRNP K to one CCC column of the other opposite hairpin and disrupting the hydrogen bonds. Because one column was stripped, the distance between the two ends of this unfolded i-motif structure was predicted to be greater than in the well-folded state, leading to a lower FRET efficiency of Cy3 and Cy5. To confirm this hypothesis, one CCC column was replaced by TTT to mimic this intermediate structure, termed Py25(245)′, which can fold into one opposite hairpin at most (Fig. 6*A*). When the pH value was less than 5.8 (Fig. 6*B*), the FRET distributions consisted of three Gaussian peaks at ∼0.4, ∼0.6, and ∼0.7 (Fig. 6, *C* and *D*, left panel), in agreement with the state transitions shown in the corresponding TDPs (Fig. 6, *C* and *D*, right panel) and representative traces (Figs S6, *A* and *B*). This finding supported the possibility that the ∼0.7 state was an opposite hairpin, and the missing FRET ∼0.9 state further supported that FRET ∼0.9 represented well-folded i-motif structures (Figs. 3*B* and 5*C*).

**Figure 7:**
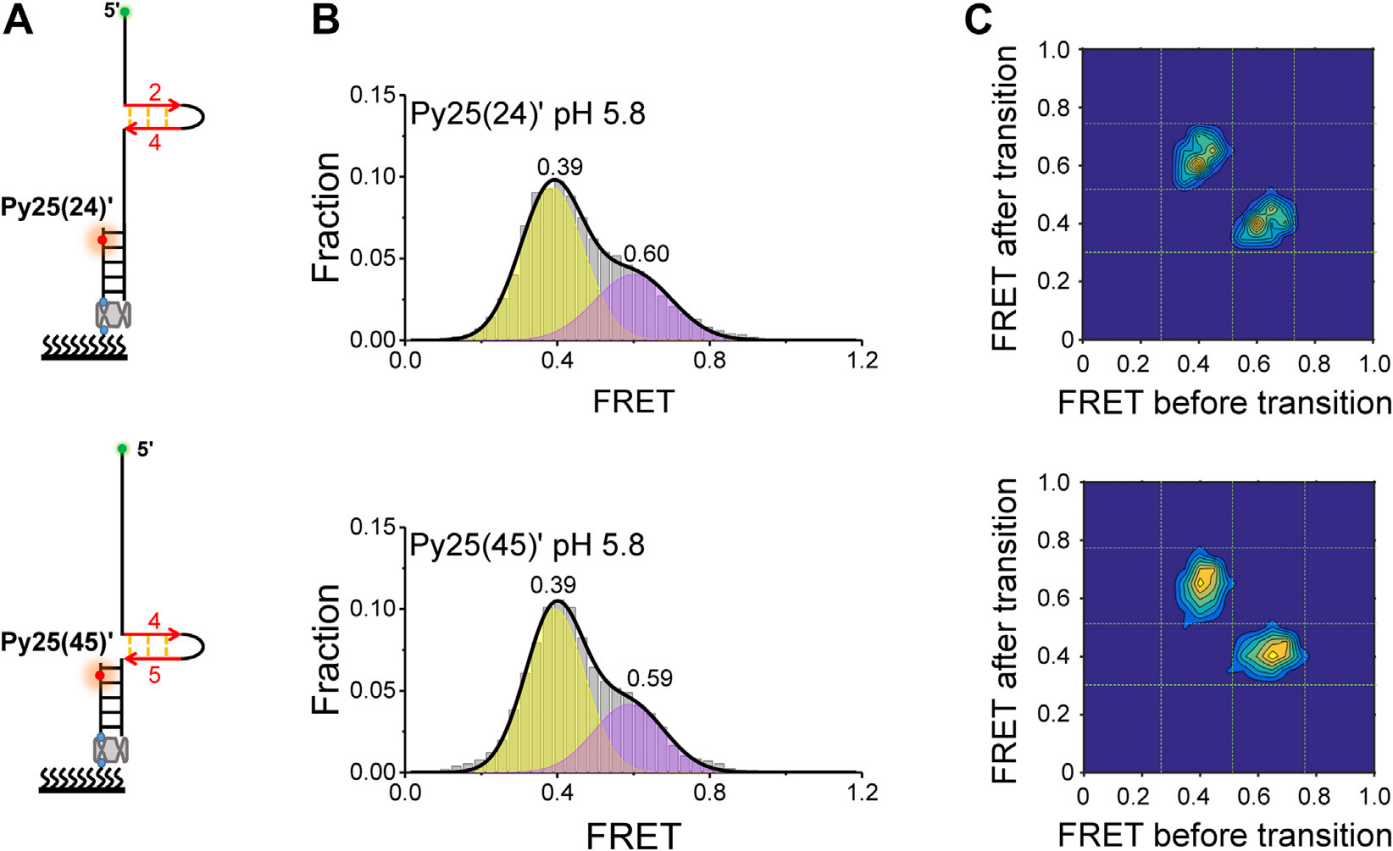
**Formation of neighboring C-hairpins at pH 5.8.***A*, schematic diagram of Py25(24)′ (*top panel*) and Py25(45)′ (*bottom panel*). *B*, FRET distributions of Py25(24)′ (*top panel*) and Py25(45)′ (*bottom panel*). Multiple-peak Gaussian distributions were used to fit their histograms. *C*, transition density plots (TDPs) of Py25(24)′ (*top panel*) and Py25(45)′ (*bottom panel*).

**Figure 8:**
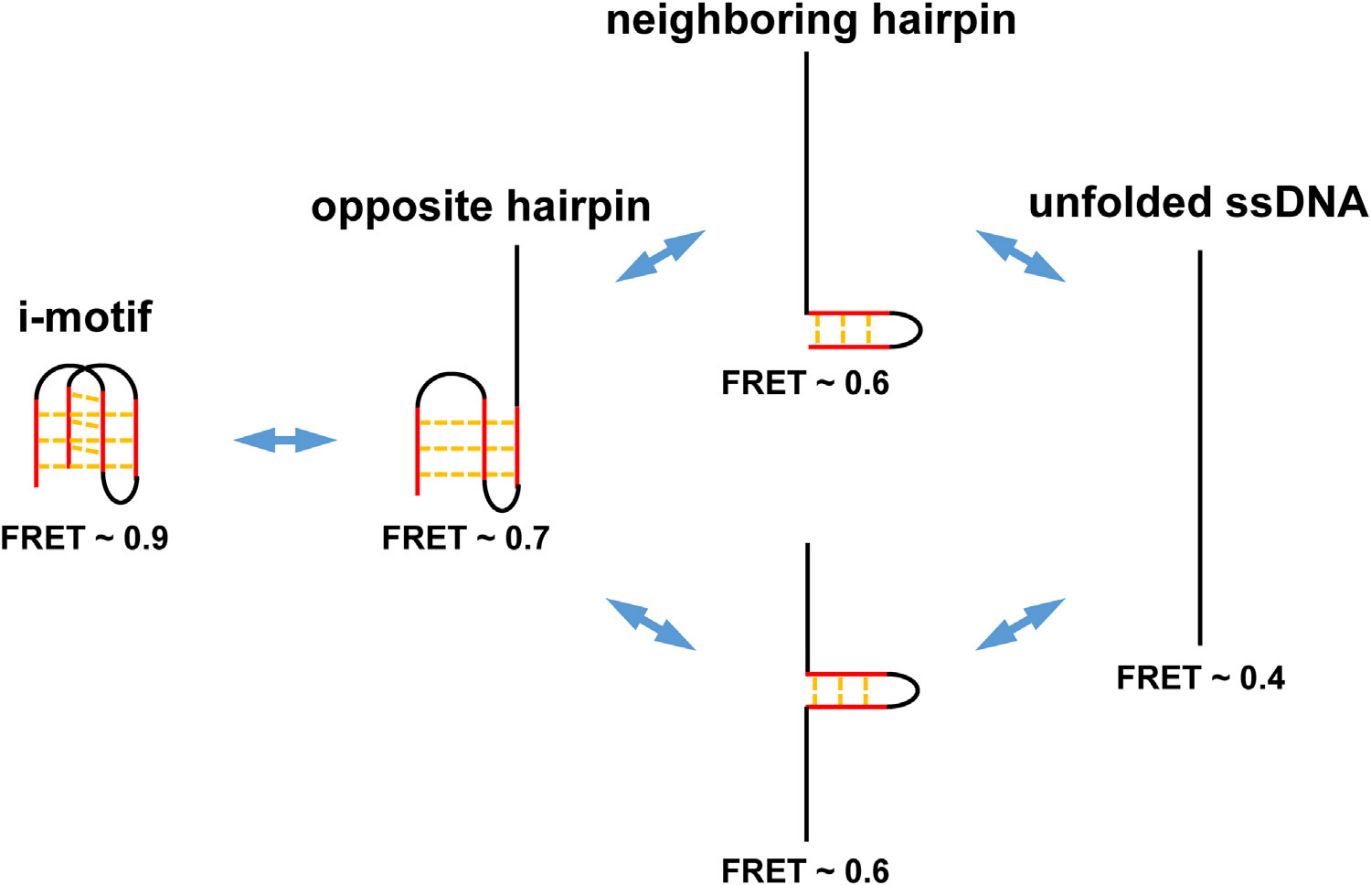
**Proposed pathways of hnRNP K unfolding i-motif DNA.** The resolving processes include well-folded i-motif structures, opposite hairpins, neighboring hairpins, and unfolded ssDNA, corresponding to the FRET values of ∼0.9, ∼0.7, ∼0.6, and ∼0.4, respectively. Their state transitions are indicated by two-way arrows. hnRNP K, heterogeneous nuclear ribonucleoprotein K.

Further, this study aimed to determine what the ∼0.6 state was assigned to. This state was found in both hnRNP K unfolding Py25′ (Fig. 5) and the dynamics of Py25(245)′ (Fig. 6). In addition to opposite hairpins, poly(C) runs may also fold into neighboring hairpins, which may form when one column of the remaining opposite hairpin is further trapped by a KH domain. It was speculated that the ∼0.6 state should consist of neighboring hairpins. To verify this speculation, this study further mutated one column of the second opposite hairpin (Fig. 6*A*) and obtained two substrates that were termed Py25(24)′ and Py25(45)′ (Fig. 7*A*). Because one more column was stripped, the two ends of the neighboring-hairpin state should be further apart than observed in the opposite-hairpin state (Fig. 7*A* *versus* Fig. 6*A*), resulting in a lower FRET value. As expected, at pH 5.8, both of these substrates could fold into two states at ∼0.4 and ∼0.6 (Fig. 7*B*), consistent with the state transitions shown in the corresponding TDPs (Fig. 7*C*) and representative traces (Fig. S6, *C* and *D*). Because Py25(24)′ and Py25(45)′ can only fold into neighboring hairpins, this result confirmed the state of FRET ∼0.6 was a neighboring hairpin, and the missing ∼0.7 state was further demonstrated to be an opposite hairpin. As the hnRNP K concentration increased, the two opposite hairpins of the i-motif were gradually resolved, and the neighboring hairpins had an opportunity to form, resulting in a gradual decrease in the proportion of the ∼0.7 state (I2) and a gradual increase in the proportion of the 0.6 state (I1) (Fig. 5, *C*–*E*). Therefore, this state assignment was reasonable. In addition, the state transitions between opposite hairpins and neighboring hairpins were captured directly from TDPs (Figs. 5*D*, 6*C* and *D*). The intermediate states were also directly captured in Py25′ at pH 6.1 (Fig. S7). Therefore, i-motif DNA-unfolding pathways involving opposite and neighboring hairpins were proposed (Fig. 8).

In previous studies, one (12, 13, 15) or four (14) folding-unfolding intermediate states were detected during i-motif thermodynamic fluctuations. However, the findings of the present study clearly demonstrated that there were two intermediates during the hnRNP K unfolding of the *c-MYC* i-motif structure. In addition, the intermediates were assigned to the specific opposite and neighboring hairpins. Therefore, there should be a difference between thermodynamic fluctuations and protein-assisted unfolding.

### All three KH domains are involved in the i-motif unfolding

**Figure 9:**
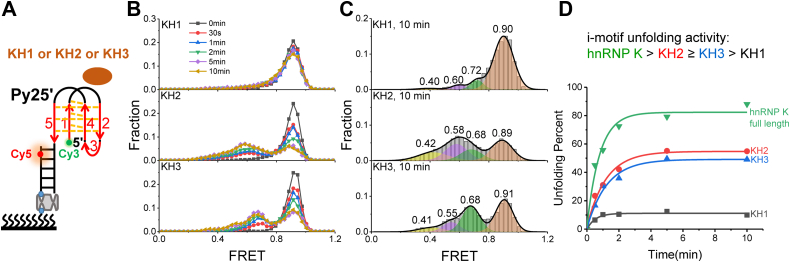
**All three KH domains can unfold i-motif DNA.***A*, schematic diagram of KH1-3 and Py25′ i-motif DNA. *B*, single-molecule FRET (smFRET) histograms obtained from adding 1 μM KH1 (*top panel*), KH2 (*middle panel*), and KH3 (*bottom panel*) at series time at pH 5.8. *C*, multipeak Gaussian distributions were used to fit the smFRET histograms at 10 min, showing four peaks. The peak values are inserted in the corresponding figures. *D*, resolving fractions of Py25′ i-motif DNA at different times after the addition of 1 μM proteins. Single-exponential decays were used to fit the resolving fractions. The parameters are shown in Table S3.

hnRNP K contains three conserved KH domains (Fig. S1*A*) that play a key role in i-motif unfolding. However, whether all three KH domains have unfolding abilities and which KH domain is the most active remain unclear. To answer these questions, each KH domain was expressed and purified (Fig. S1*C*), and 1 μM of each KH domain was separately incubated with Py25′ (Fig. 9*A*). It was clear that the proportion of the ∼0.9 peaks gradually decreased over time, indicating that all three KH domains were able to unfold the Py25′ i-motif structure (Fig. 9*B*). In addition, their FRET histograms were also well fitted to four states using a multipeak Gaussian distribution (Fig. 9*C*). The FRET values were close to the above fitting, further indicating that the proposed model was reasonable (Fig. 8). To compare the activity levels of the three KH domains, the unwinding fractions of the Py25′ i-motifs calculated based on the peak areas of FRET ∼0.9 at certain times were fitted using single-exponential decays (Fig. 9*D*) (33, 35). Their unwinding parameters are shown in Table S3. Based on the comparison of their unwinding fractions and rates, it was concluded the activity of KH2 was slightly greater than that of KH3, with an ∼1.1-fold increase in unfolding amplitude and rate, while both were significantly more active than KH1, based on the ∼4-fold and ∼2-fold increases in amplitude and unwinding rate, respectively. In addition, the activity of any single KH motif was less than that of the full-length hnRNP K under the same experimental conditions (Fig. 5*B*, bottom panel); for example, KH1 and KH2 activity levels were approximately half that of the full length hnRNP K in terms of unwinding amplitude and rate. From another perspective, at 10 min, the interaction between each KH motif and the Py25′ i-motif could be fitted to four peaks (Fig. 9*C*), while there were only three peaks for hnRNP K (Fig. 5*C*, bottom panel), where the ∼0.7 peak corresponding to the opposite hairpin disappeared, further indicating that hnRNP K was more active.

## Discussion

The structural dynamics of i-motifs play a regulatory role in numerous life processes. Therefore, studying the molecular mechanisms of how proteins unfold i-motif structures is fundamentally essential. However, this process is not fully understood. This work selected the interaction between hnRNP K and *c-MYC* i-motif as an example to reveal the molecular dynamic details of how PCBPs unfold i-motif structures at the single-molecule level and proposed the unfolding pathway. In addition, the i-motif–unwinding abilities of all three KH domains of hnRNP K were explored. The results of this study should be useful in understanding the cellular mechanisms of i-motif dynamics and aid in drug design.

After verifying the formation of i-motif structures by CD (Fig. 1), the type-1245 structure folded from Py25(1245) was proposed to be the major contributor to Py25 sequences based on *T*_m_ comparison (Fig. 2). This proposal was further confirmed by the consistency of FRET values at the single-molecule level (Fig. 3). In addition, hnRNP K resolving Py25′ and Py25(1245)′ exhibited similar phenomena, further supporting the possibility that they have similar structures (Figs. 5 and S4). Therefore, it was concluded that Type-1245 was the main i-motif structure of Py25. This series of methods may be used to determine the major folded structures of other substrates.

The ability of hnRNP K to unfold the *c-MYC* i-motif structure was confirmed by a bulk FRET assay (Fig. 4). Using the smFRET technique, this work first reported that hnRNP K resolved i-motif DNA discretely with opposite hairpins and neighboring hairpins as intermediate states (Fig. 5), which was further verified by substrate mutations (Figs. 6 and 7), and the unfolding pathway was proposed (Fig. 8). In addition, individual KH domain unwinding experiments further supported this pathway (Fig. 9*C*). These details not only deepen the understanding of how PCBPs unfold i-motif structures but may also provide a reference for how other proteins resolve i-motif DNA. The step-by-step process of hnRNP K resolving the *c-MYC* i-motif may provide more regulatory dimensions in cellular processes and may be useful in drug design. Interestingly, G-quadruplexes are also reported to contain two kinds of intermediates during unfolding, which are G-triplex and G-hairpin (36, 37). Recently, research based on structural biology revealed the existence of the C-triplex (38), which could not be ruled out from the opposite hairpin in the present experiments because of their similar FRET values. Whether C-triplex is an intermediate state for i-motif unwinding deserves further study.

Finally, the present study analyzed the i-motif–unfolding ability of all three KH domains (Fig. 9). It was found that all three KH domains could resolve Py25′ i-motifs with activity levels that ranked as follows: KH2 ≥ KH3 > KH1 (Fig. 9*B*). However, the KH domains were less active than full-length hnRNP K (Fig. 9*D*). It has been reported that only KH3 of hnRNP K can bind poly(C) RNA, while KH1 and KH2 cannot (39). Therefore, our future work will focus on revealing the differences between hnRNP K unfolding i-motif DNA and RNA at the single-molecule level.

## Experimental procedures

### Buffers

All of the reaction buffers contained 50 mM PBS at the corresponding pH, 200 mM K^+^ (final concentration, supplemented with KCl), 40% PEG200 (w/v), and 4 mM Trolox. For single-molecule experiments, the oxygen scavenging system (0.8% D-glucose, 1 mg/ml glucose oxidase (266600 units/g, Sigma), and 0.4 mg/ml catalase (2000–5000 units/mg, Sigma) were added.

### Substrate preparation

All of the DNA substrates used in this study are listed in Table S1. For the DNA preparation of CD experiments, the final concentration of annealed ssDNA was 10 μM. For the substrate preparation of ensemble FRET experiments, the ratio of Cy3-labeled ssDNA and Cy5-labeled ssDNA was 1:1 to ensure that the fluorescence intensity directly reflected the structural states, and the concentration was 2 μM. For single-molecule experiments, the ratio of biotin-modified ssDNA to biotin-free ssDNA was 5:7 annealed to prevent the possibility that nonannealed ssDNA might anchor to the coverslip surface. The final concentration of biotin-modified ssDNA was 2 μM. All of the annealing was performed by incubation at 95 °C for 5 min and then slowly cooled to 20 °C in the corresponding reaction buffers.

### CD spectropolarimetry

CD experiments were performed on a Chirascan V100 (Applied Photophysics Ltd) that contained a temperature probe and a temperature-controlled cell holder (32). The CD signals were recorded in the 220 to 340 nm region using a quartz cell (0.5-mm path length). The melting curves were determined between 20 to 98 °C, and the temperature rose one degree per minute. The *T*_m_ values were calculated using the analysis software supplied with the instrument.

### Protein expression and purification

Full-length hnRNP K and fragments of KH1 (hnRNP K^1-127^), KH2 (hnRNP K^127-237^), and KH3 (hnRNPK^382-463^) were expressed and purified (Fig. S1). The corresponding genes were cloned into pET21a using the *Nde*I and *Xho*I sites. For KH1 and KH3, GST tags were fused to their N-terminal to improve solubility. Each expression vector was transformed into *Escherichia coli* Rosetta2 (DE3). Expression strains were incubated in LB at 37 °C before the A_600_ reached about 0.7. Protein expression was induced with 0.3 mM IPTG at 16 °C overnight. After cell lysis, proteins were captured by Ni-NTA beads. The fused GST tag was removed by PreScission Protease, if applicable. Gel-filtration chromatography was subsequently carried out to polish proteins. All of the purified proteins were frozen in small aliquots and stored at -80 °C after being fast-frozen in liquid nitrogen. The purity of the proteins was more than 95%, as shown in Fig. S1 by SDS-PAGE.

### DNA-binding assay

Different concentrations of hnRNP K were mixed with 80 nM fluorescently labeled Py25′ in reaction buffer (pH 5.8). The mixture was electrophoresed on an 8% native PAGE after being incubated for 20 min at 22 °C. The gels were run in TAE buffer containing 200 mM KCl and imaged on a ChemiDoc MP Visualization System (BioRad); the images were analyzed in ImageJ (National Institutes of Health).

### Ensemble FRET

The bulk FRET experiments were carried out on a homebuilt optical system. The final concentration of 4 nM i-motif–contained substrate and different concentrations of hnRNP K were mixed in a quartz cell with a path length of 5 mm at a constant temperature of 20 °C, and a laser set at 532 nm was used to excite Cy3. The emission of optical signals at 550 to 800 nm was recorded on an Avantes Fluorescence Spectrometer LS55 at a certain time.

### smFRET

The equipment, procedure, and data analysis were the same as reported in this study group’s previous work (32, 33). Briefly, all smFRET experiments were conducted using a homebuilt objective-type total-internal-reflection microscope at 22 °C. The reaction chambers consisted of slides and coverslips. The surfaces of the coverslips were coated with a mixture of 99% mPEG (m-PEG-5000, Laysan Bio, Inc) and 1% of biotin-PEG (biotinPEG-5000, Laysan Bio, Inc). Streptavidin was used to capture biotin-modified substrates and immobilize them to the coverslips through biotin-streptavidin conjunction. The oxygen scavenging system was used to prevent bleaching and blinking. The FRET values were calculated using *I*_A_/(*I*_D_ + *I*_A_), where *I*_D_ and *I*_A_ represent the fluorescence intensity of the donor (Cy3) and acceptor (Cy5), respectively. The exposure time for imaging was 100 ms, and the reaction temperature was 22 °C. FRET histograms were obtained by collecting 50 frames of each trace from more than 300 molecules. The FRET distributions of different states were recognized and fitted using a multipeak Gaussian distribution by OriginPro 2017 (14, 32, 34). More than 200 traces containing state transitions were selected to construct TDPs following the previously reported method (33).

## Data availability

All data are contained within the article.

## Supporting information

This article contains supporting information.

## Conflict of interest

The authors declare that they have no conflicts of interest with the contents of this article.
