# Supplementary material for: Mechanistic insights into poly(C)-binding protein hnRNP K resolving i-motif DNA secondary structures
Source: J Biol Chem. 2022 Nov 2;298(12):102670. doi: 10.1016/j.jbc.2022.102670 (PMC9709238; doi:10.1016/j.jbc.2022.102670)
Supplement: Supplementary Data [file mmc1.pdf]

Supporting Information

Mechanistic insights into poly(C)-binding protein hnRNP K resolving i-motif DNA secondary structures

Wen-Qiang Wu[†,*], Xin Zhang[†], Di Bai, Song-Wang Shan, Li-Jun Guo[*]

From School of Life Sciences, Academy for Advanced Interdisciplinary Studies, State Key Laboratory of Crop Stress Adaptation and Improvement, School of Physics and Electronics, Henan University, Kaifeng 475001, China.

[†]These authors contributed equally to this work as Joint First Authors.
[*]To whom correspondence should be addressed.

E-mail: wuwenqiang@henu.edu.cn (W.-Q. W), juneguo@henu.edu.cn (L.-J. G)

Tables S1-S3

Figures S1-S7

Table S1. Sequences of substrates used in the experiments.

**Name**	**For figures**	**Sequences (5'-3') of substrates for CD**
Py25	Figure 1	TCCCCACCCTCCCCACCCTCCCC
Py25(1245)	Figures 1 and 2	TCCCCACCCTTTTTACCCTCCCC
Py25(1234)	Figures 1 and 2	TCCCCACCCTCCCCACCCTTTTT
		**Sequences (5'-3') of substrates for FRET**
Py25'	Figures 3, 4, 5, 9, S2, S3, S5 and S7	TTCCCCACCCTCCCCACCCTCCCCATGAGGACACGTGCATTCC<br>Biotin-GGAATGCACGTGTCCTC
Py25(1245)'	Figures 3, S2, S4 and S5	TTCCCCACCCTTTTTACCCTCCCCATGAGGACACGTGCATTCC<br>Biotin-GGAATGCACGTGTCCTC
Py25(1234)'	Figures 3 and S2	TTCCCCACCCTCCCCACCCTTTTTATGAGGACACGTGCATTCC<br>Biotin-GGAATGCACGTGTCCTC
Py25(1235)'	Figure S2	TTCCCCACCCTCCCCATTTTCCCCATGAGGACACGTGCATTCC<br>Biotin-GGAATGCACGTGTCCTC
Py25(1345)'	Figure S2	TTCCCCATTTTCCCCACCCTCCCCATGAGGACACGTGCATTCC<br>Biotin-GGAATGCACGTGTCCTC
Py25(2345)'	Figure S2	TTTTTTACCCTCCCCACCCTCCCCATGAGGACACGTGCATTCC<br>Biotin-GGAATGCACGTGTCCTC
Py25(245)'	Figures 6 and S6	TTTTTTACCCTTTTTACCCTCCCCATGAGGACACGTGCATTCC<br>Biotin-GGAATGCACGTGTCCTC
Py25(45)'	Figures 7 and S6	TTTTTTATTTTTTTTACCCTCCCCATGAGGACACGTGCATTCC<br>Biotin-GGAATGCACGTGTCCTC
Py25(24)'	Figures 7 and S6	TTTTTTACCCTTTTTACCCTTTTTATGAGGACACGTGCATTCC<br>Biotin-GGAATGCACGTGTCCTC

Color: Green, Cy3; Red, Cy5. **Bold**: **C sequences**. Underline: dsDNA forming sequences.

Table S2. Kinetic parameters of hnRNP K resolving the Py25' i-motif at different protein concentrations using bulk fluorescence spectra obtained from the exponential fittings of the data shown in Figure 4C.

Concentration	Unwinding amplitude of Cy3 (a.u.)	Rate constant (min^{-1})	Unwinding rate (a.u. min^{-1})
4 nM	1451.83 ± 270.29	0.15 ± 0.06	234.63 ± 126.82
20 nM	2128.24 ± 183.29	0.44 ± 0.10	947.84 ± 287.51
40 nM	2297.63 ± 319.21	0.46 ± 0.16	1115.41 ± 524.77
200 nM	10134.70 ± 395.94	0.36 ± 0.04	3644.02 ± 525.37
400 nM	10781.06 ± 618.93	0.51 ± 0.07	5605.80 ± 1113.23
2 μM	16311.28 ± 359.88	0.95 ± 0.05	15547.74 ± 1162.93
4 μM	16337.09 ± 1007.81	1.45 ± 0.25	23869.24 ± 5579.11

Table S3. Kinetic parameters of KH1-3 and hnRNP K resolving the Py25' i-motif at 1 μM protein, as obtained from exponential fittings of the data in Figure 9D.

Protein	Unfolding percent	Rate constant (min^{-1})	Unwinding rate (min^{-1})
KH1	11.22 ± 1.32	1.93 ± 0.55	22.34 ± 8.74
KH2	53.11 ± 3.27	0.84 ± 0.12	44.88 ± 9.27
KH3	48.46 ± 3.13	0.80 ± 0.12	39.18 ± 8.53
hnRNP K	80.48 ± 6.68	1.25 ± 0.24	101.94 ± 27.57

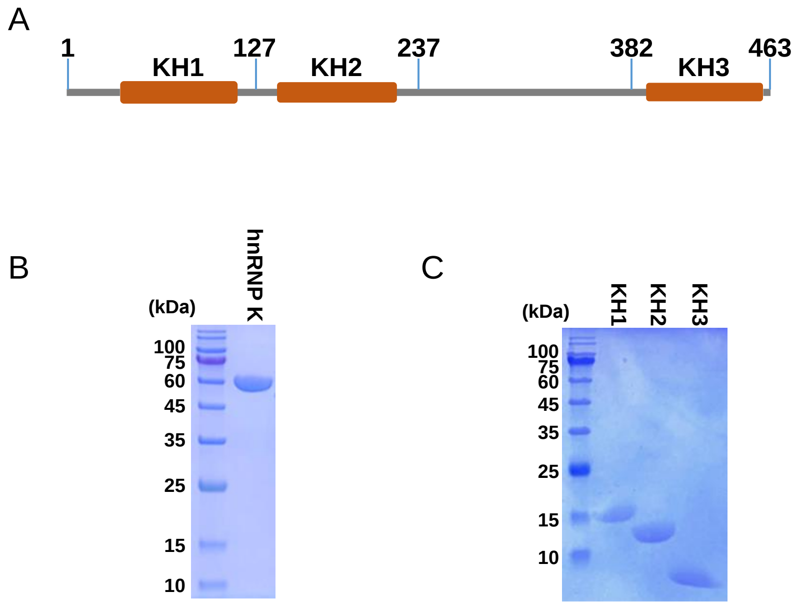

Figure S1. Domain architecture and purification. (**A**) Diagram of hnRNP K. (**B**, **C**) Analysis of the purified hnRNP K (B) and KH1-3 (C) by SDS-PAGE.

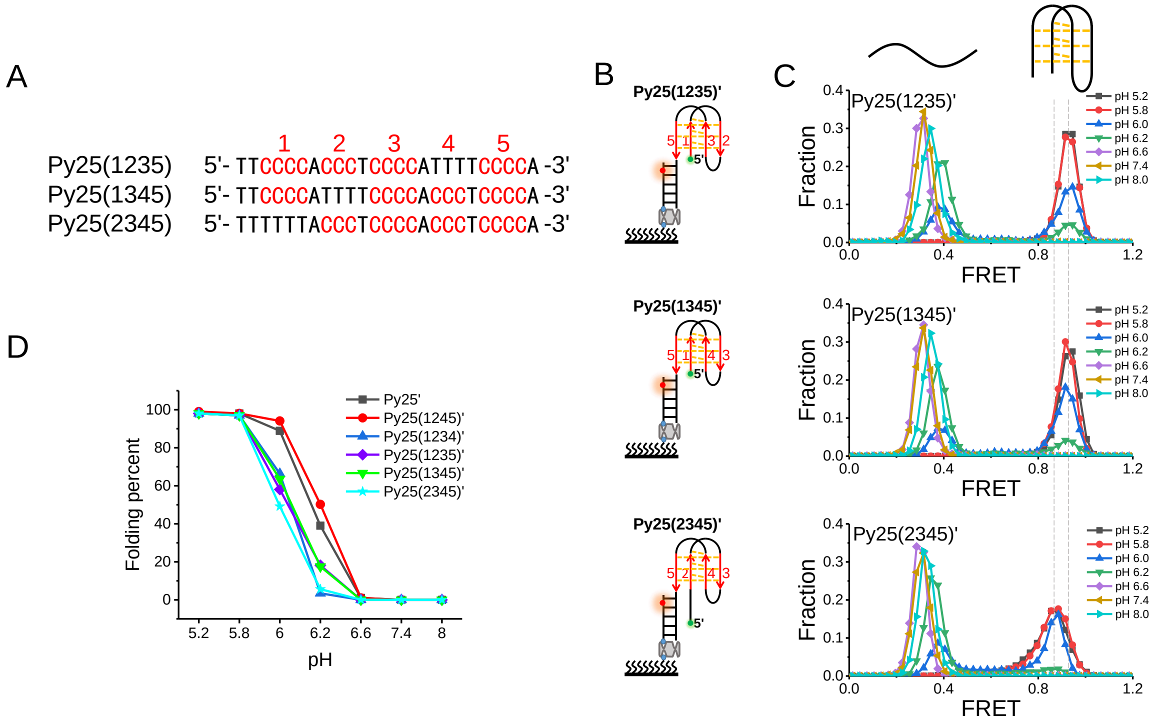

Figure S2. Folding of Py25(1235)', Py25(1345)', and Py25(2345)' at different pH levels from 5.2 to 8.0 using smFRET. (**A**) The sequences of Py25(1235), Py25(1345), and Py25(2345). (**B, C**) Schematic diagram and the FRET distributions at different pH of Py25(1235)', Py25(1345)', and Py25(2345)'. FRET values below 0.4 indicate ssDNA, and FRET values exceeding 0.8 indicate unfolded i-motif DNA (Figure 3). (**D**) Folding percentages of Py25', Py25(1245)', Py25(1234)', Py25(1235)', Py25(1345)', and Py25(2345)' at different pH values from 5.2 to 8.0, as derived from Figure 3 and (C).

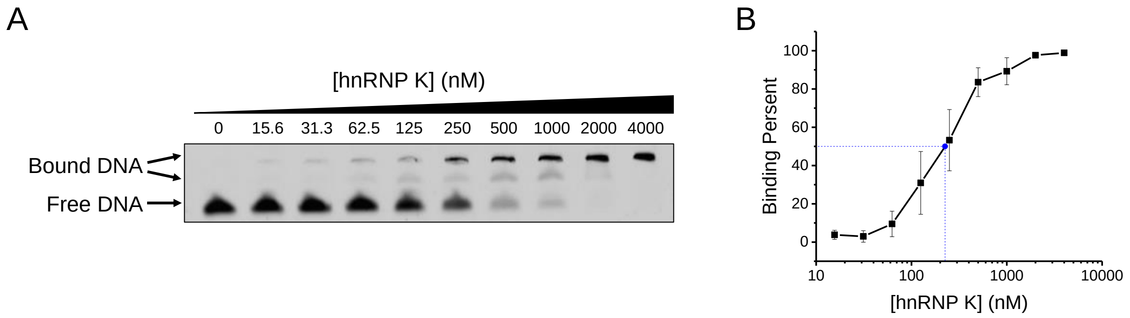

Figure S3. The binding of hnRNP K with Py25'. (**A**) Different concentrations of hnRNP K were mixed with 80 nM Py25', and PAGE bandshift assays determined the binding. (**B**) Quantitative analyses of hnRNP K binding Py25' by ImageJ (n = 3). The apparent DNA binding affinity value was approximately 220 nM.

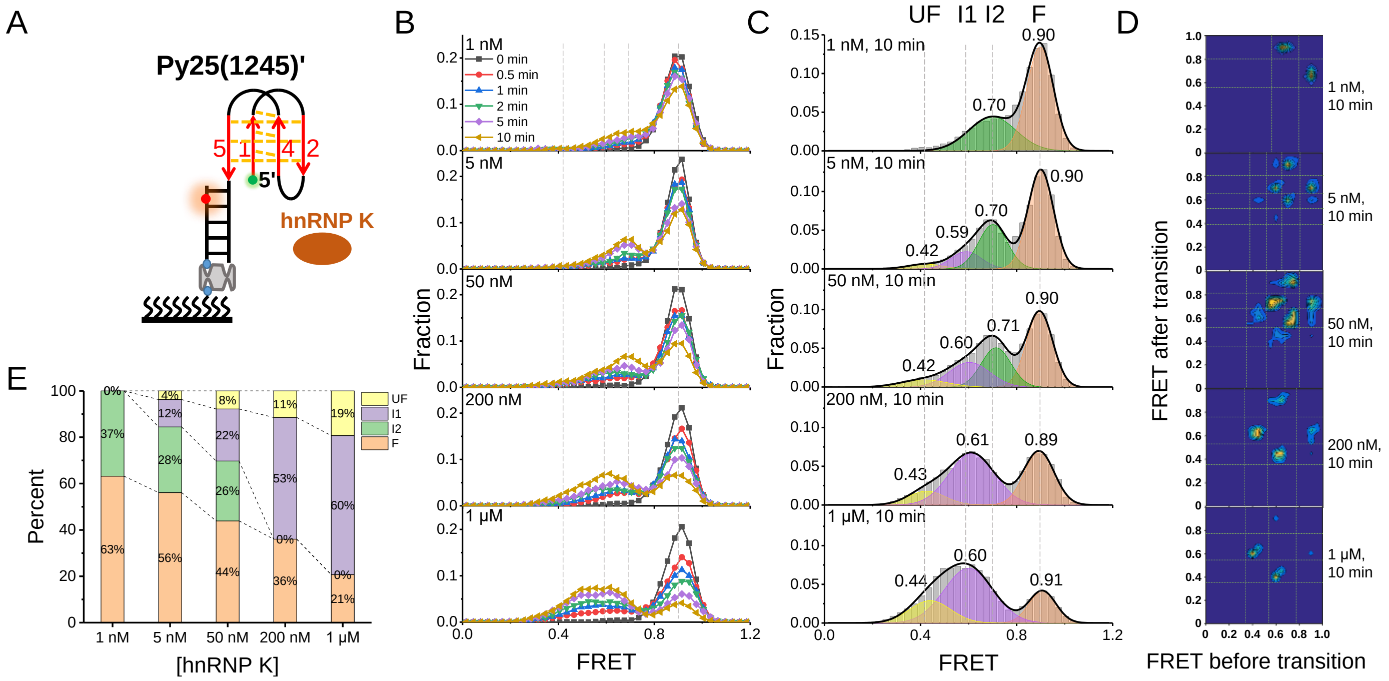

Figure S4. hnRNP K unfolding type-1245 i-motif DNA discretely revealed by single-molecule fluorescence resonance energy transfer (smFRET). (**A**) Schematic diagram of hnRNP K and Py25(1245)' i-motif DNA. (**B**) smFRET histograms obtained by adding 1 nM–1 μM hnRNP K at series times from 0 to 10 min at pH 5.8. (**C**) Multipeak Gaussian distributions were used to fit the smFRET histograms at 10 min. The peak values are shown in the corresponding figures. (**D**) Transition density plots (TDPs) were used to show the state transitions of hnRNP K unfolding Py25(1245)' at 10 min and certain concentrations. (**E**) The fractions of the different folding structures at increasing concentrations of hnRNP K. UF, unfolded state; I1, intermediate state 1; I2, intermediate state 2; F, folded state.

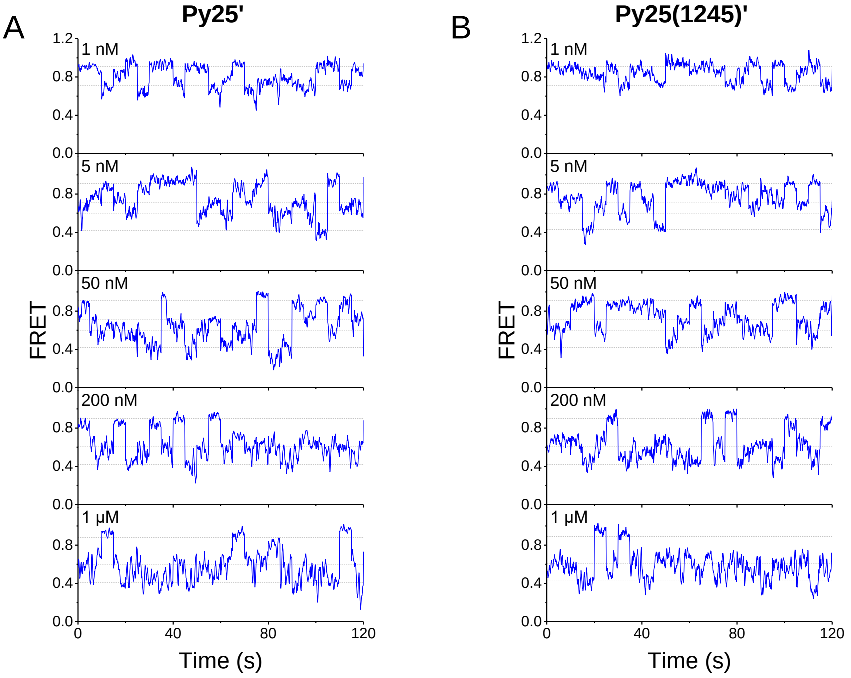

Figure S5. Representative single-molecule fluorescence resonance energy transfer (smFRET) traces of Py25' and Py25(1245)' measured at different concentrations of hnRNP K at pH 5.8.

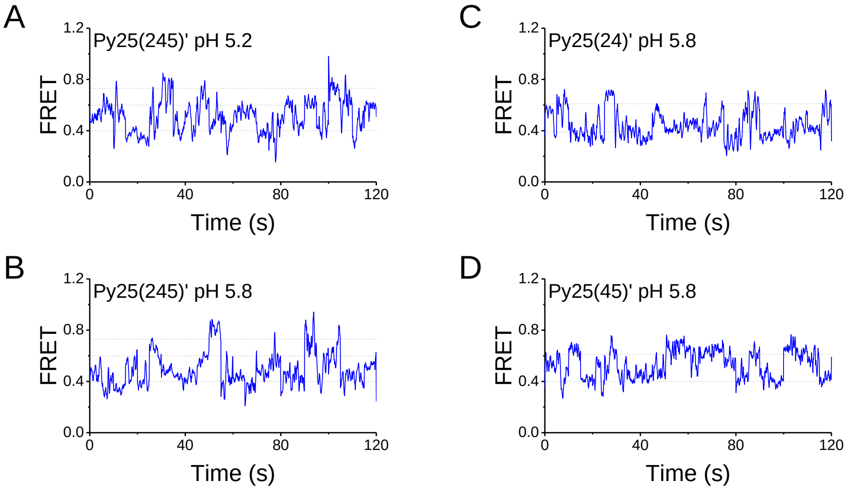

Figure S6. Representative single-molecule fluorescence resonance energy transfer (smFRET) traces of Py25(245)', Py25(24)', and Py25(45)' measured at corresponding pH values.

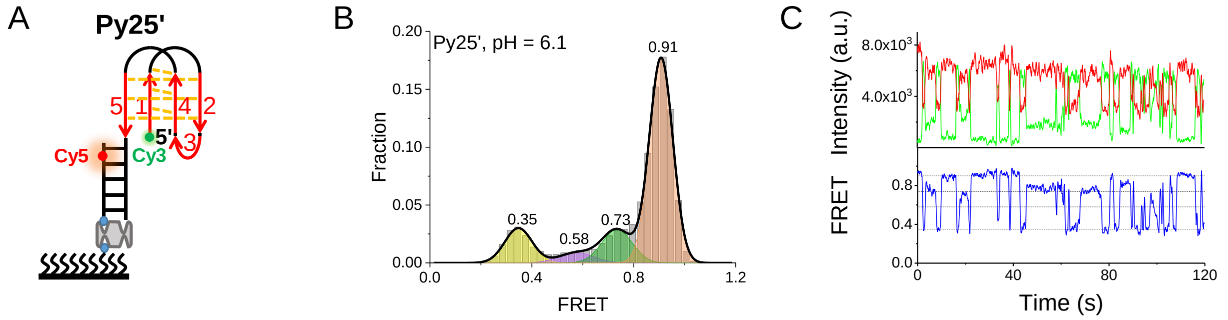

Figure S7. The folding of Py25' at pH 6.1. (**A**) Schematic diagram of Py25'. (**B**) FRET distributions of Py25' at pH 6.1. Multipeak Gaussian distributions were used to fit the smFRET histogram. (**C**) Representative time traces of fluorescence intensities of Cy3 and Cy5 (upper panel) and the corresponding FRET trace (lower panel).
